# Altered isotropic volume fraction in gray matter after sleep deprivation and its association with visuospatial memory: A neurite orientation dispersion and density imaging study

**DOI:** 10.3389/fnins.2023.1144802

**Published:** 2023-03-23

**Authors:** Jia-Hui Lin, Xu-Hui Chen, Ye Wu, Yun-Bin Cao, Hua-Jun Chen, Nao-Xin Huang

**Affiliations:** ^1^Department of Radiology, Fujian Medical University Union Hospital, Fuzhou, China; ^2^School of Computer Science and Engineering, Nanjing University of Science and Technology, Nanjing, China

**Keywords:** sleep deprivation, neurite orientation dispersion and density imaging, gray matter, isotropic volume fraction, visuospatial memory

## Abstract

**Background and aims:**

Diffusion magnetic resonance imaging (dMRI) studies have revealed microstructural abnormalities in white matter resulting from sleep deprivation (SD). This study aimed to adopt neurite orientation dispersion and density imaging (NODDI) to investigate the effect of SD on gray matter (GM) microstructural properties and its association to visuospatial memory (VSM).

**Methods:**

Twenty-four healthy women underwent two sessions of dMRI scanning and visuospatial ability assessment by Complex Figure Test (CFT), once during rested wakefulness (RW) and once after 24 h of SD. We calculated NODDI metrics, including intracellular volume fraction (ICVF), orientation dispersion index (ODI), and isotropic volume fraction (ISO). Differences in NODDI-related metrics between RW and SD were determined using a voxel-wise paired *t-*test. We identified an association between NODDI metrics and CFT results using Spearman’s correlation coefficient.

**Results:**

Sleep deprivation worsened subjects’ performance in the delayed-CFT trial. We observed no significant difference in ICVF and ODI between RW and SD. After SD, subjects showed decreases in ISO, primarily in the prefrontal cortex and temporal lobe, while exhibiting ISO increases in the anterior and posterior cerebellar lobe and cerebellar vermis. Furthermore, ISO change in the left superior, middle and inferior frontal gyrus was significantly correlated with completion time change in delayed-CFT trial performance.

**Conclusion:**

Our results suggested that SD hardly affected the density and spatial organization of neurites in GM, but the extra-neurite water molecule diffusion process was affected (perhaps resulting from neuroinflammation), which contributed to VSM dysfunction.

## Introduction

Sleep is a complex physiological process that is essential in humans for maintaining cognition (Colrain, 2011). Visuospatial memory (VSM) is one of the crucial domains in cognition that plays a basic role in daily life, which is required for orientation, spatial localization, and using a navigation map (Perrochon et al., 2014). Sleep deprivation (SD), even a single night thereof, can have detrimental effects on VSM (Gosselin et al., 2017; Pasula et al., 2018). Meanwhile, animal experiments have found that SD is associated with alterations in brain structure. For example, one study revealed that SD increases the breakdown of neuronal-membrane phospholipids (Hinard et al., 2012). Another study found that SD induces spine density changes in the hippocampus and prefrontal cortex in rats (Acosta-Pena et al., 2015). However, the neural mechanisms underlying VSM deficit after SD are not well understood. Elucidating the neurobiological effects of SD on the brain might help us grasp the significance of sleep in neuroscience.

Diffusion magnetic resonance imaging (dMRI) is a non-invasive approach that measures the diffusion of water molecules in tissue, providing information about the underlying brain microstructure (Pierpaoli et al., 1996). In recent years, many studies have used dMRI to investigate the effect of SD on brain microstructure (Rocklage et al., 2009; Cui et al., 2015; Elvsashagen et al., 2015; Zhu et al., 2017; Voldsbekk et al., 2021; Wang et al., 2022). For instance, one diffusion tensor imaging (DTI) study found that SD was associated with impaired white matter (WM) integrity [as reflected by reduced fractional anisotropy (FA)] of the bilateral frontotemporal and parieto-occipital tracts, corpus callosum, thalamus, and brain stem (Elvsashagen et al., 2015). In addition, several DTI studies have reported that WM microstructural properties enable the prediction of cognitive vulnerability to SD (Rocklage et al., 2009; Wang et al., 2022). Moreover, the individual’s cognitive stability/resistance to SD is associated with the integrity of the WM tract, which connects the frontoparietal attention networks (Cui et al., 2015; Zhu et al., 2017). Notably, several inherent drawbacks of DTI limit its utility in SD-related studies. For example, DTI assumes that water diffusion is Gaussian distribution, so it is not able to completely characterize tissue microstructure (Steven et al., 2014). In addition, DTI extracts information from dMRI data *via* “signal representations” approach, which lacks specificity and remains an indirect characterization of microstructure (Huang S. et al., 2022). Furthermore, due to its assumption of a single tissue compartment, DTI cannot distinguish microstructural properties between intra-cellular and extra-cellular spaces (Beaulieu, 2002).

In recent years, several advanced multi-compartment diffusion models have been proposed to overcome the limitations of DTI. For instance, a recent dMRI study using spherical mean technique (SMT), a multi-compartment model that estimates brain tissue microstructure in the intra- and extra-axonal spaces, revealed that the effect of SD on WM microstructure mainly involves the extra-axonal water molecule diffusion process (Voldsbekk et al., 2021). Neurite orientation dispersion and density imaging (NODDI) is another multi-compartment diffusion model that parametrizes the dMRI signal as a function of biophysically meaningful parameters [e.g., dendrite and axon density in gray matter (GM) and WM, respectively] (Zhang et al., 2012; Kamiya et al., 2020), which has been validated in the histology of animal and human brain (Sepehrband et al., 2015). NODDI-derived metrics provide detailed information on the brain’s tissue microstructure. Specifically, intracellular volume fraction (ICVF) quantifies the packing density of neurites (including dendrites and axons); the orientation dispersion index (ODI) indicates the spatial organization or geometric complexity of neurites; and isotropic volume fraction (ISO) reflects extra-cellular isotropic diffusion (Kraguljac et al., 2022). The primary advantage of NODDI is that its metrics are more directly related to the brain’s microstructure because it models the biophysical properties of the tissue (Kamiya et al., 2020). NODDI has been used to explore brain microstructural alterations in several physiological and pathological conditions, such as brain development (Zhao et al., 2021), aging (Merluzzi et al., 2016), Parkinsonism (Mitchell et al., 2019), and Wilson disease (Song et al., 2018). Nevertheless, previous DTI and SMT studies have focused solely on WM microstructural changes after SD (Rocklage et al., 2009; Cui et al., 2015; Elvsashagen et al., 2015; Zhu et al., 2017; Voldsbekk et al., 2021; Wang et al., 2022), and the effect of SD on GM microstructure remains unclear.

In light of the above, in this study, we exploratorily adopted NODDI to: (1) investigate whether GM microstructural alteration occurred after 24 h of SD and (2) determine the association between changes in GM diffusion metrics and VSM alterations after SD.

## Materials and methods

### Subjects

Twenty-four healthy women participated in this study, who were all right-handed; the participants had an average age of 20 ± 0.81 years as well as an average of 13 ± 0.93 years of education. Participants who met any one of the following criteria were excluded: (1) a history of sleep disorder or any other neuropsychiatric conditions; (2) the taking of psychotropic medications; (3) chronic and severe medical illness such as heart failure, malignancies, or chronic renal failure; (4) contraindications to MRI scanning. The Ethical Committee of Fujian Medical University Union Hospital (Fuzhou, China) approved this study, and all subjects provided their informed written consent.

### Experimental procedure

**FIGURE 1:**
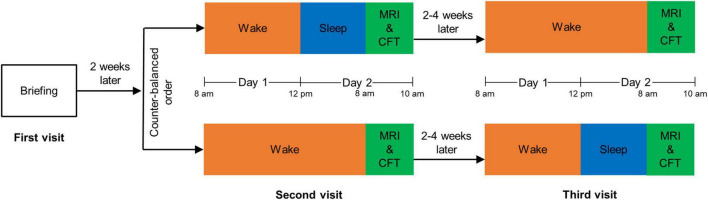
The flowchart of experimental procedure. MRI, magnetic resonance imaging; CFT, complex figure test.

We followed the same experimental procedure as that described in our previous study (Huang N. X. et al., 2022). The flowchart of experimental procedure is shown in Figure 1. Each participant made three visits to the laboratory: briefing, rested wakefulness (RW), and SD sessions. The first visit was the briefing session, in which we briefed subjects on the procedure and obtained their signed informed consent. RW and SD sessions started after at least 2 weeks of habitual sleep. To minimize the possible residual effects of SD on cognition (Van Dongen et al., 2003), the RW session and the SD session were set 2–4 weeks apart, and their order was counterbalanced across all participants.

For the RW session, we asked participants to stay awake from 8:00 a.m. to 12:00 p.m. (day 1) and to sleep from 0:00 a.m. to 8:00 a.m. (day 2). For the SD session, participants were asked to stay awake from 8:00 a.m. (day 1) to 8:00 a.m. (day 2). Subjects stayed in our laboratory for both these sessions, accompanied by two research assistants to prevent them from falling back to sleep during the awakening state. Participants were permitted to engage in non-strenuous activities such as reading, talking, or internet surfing, but strenuous activities and the intake of stimulating substances were not allowed.

### Visuospatial ability assessment

A trained staff member assessed subjects’ visuospatial ability using the Complex Figure Test (CFT) (Shin et al., 2006). CFT is a popular neuropsychological assessment tool for visuospatial ability, including three subtests: CFT-copy, immediate-CFT, and delayed-CFT trials. In the CFT-copy trial, subjects were presented the complex figure and were asked to copy the entire figure onto a blank sheet of paper; Following the CFT-copy trial, the immediate-CFT trial was performed, in which subjects were asked to reproduce the complex figure from memory; In the delayed-CFT trial that was performed 25 min after the immediate-CFT trial, subjects reproduced the complex figure once again (Shin et al., 2006). The CFT-copy trial was used to evaluate visuospatial constructional ability, while immediate-CFT and delayed-CFT trials were used to evaluate VSM (Shin et al., 2006). The assessment was scheduled for 8:00–10:00 a.m. on day 2 for each of the RW and SD sessions.

### MRI data acquisition

We acquired MRI data using a 3.0 Tesla Siemens Prisma MRI scanner (Siemens, Erlangen, Germany). Diffusion-weighted images were obtained using a multi-shell echo-planar imaging sequence, which consisted of four *b*-values (0, 1,000, 2,000, and 3,000 s/mm^2^) along 6, 30, 30, and 30 gradient directions, respectively. Other image acquisition parameters were as follows: repetition time (*T*_R_) = 4,200 ms; time to echo (*T*_E_) = 72 ms; number of averages = 1; flip angle = 90°; slice thickness = 2 mm; field of view (FOV) = 216 mm × 216 mm; matrix = 108 × 108; voxel size = 2 × 2 × 2 mm^3^; slice number = 72 (axial slices without gaps); multiband factor = 2. T1-weighted (T1W) structural images were acquired using a magnetization-prepared rapid gradient-echo (MPRAGE) sequence with the following parameters: *T*_R_ = 1,610 ms; *T*_E_ = 2.25 ms; flip angle = 8°; slice thickness = 1.0 mm; FOV = 224 mm × 224 mm; matrix = 224 × 224; voxel size = 1 × 1 × 1 mm^3^; slice number = 176 (sagittal slices without gaps). MRI scanning was scheduled for 8:00–10:00 a.m. on day 2 for each of the RW and SD sessions.

### MRI data pre-processing

We visually inspected the T1W and dMRI images of all subjects to detect any signal dropouts or artifacts. Next, we pre-processed the images *via* the well-established pipeline, described as follows. For both T1W and dMRI data, the procedure began with axial alignment, centering, Gibbs ringing removal based on Local Subvoxel-Shifts (Kellner et al., 2016), and intensity inhomogeneity correction *via* N4ITK (Tustison et al., 2010). For dMRI data, we also included the following steps: (1) Marchenko–Pastur principal-component analysis (MP-PCA) denoising (Veraart et al., 2016a,b) to improve the signal-to-noise ratio (SNR) without reducing spatial resolution; (2) FSL’s eddy_correct tool was used for eddy current correction (Jenkinson et al., 2012); (3) brain mask generation using a brain extraction tool (BET) (Jenkinson et al., 2012); and (4) distortion correction *via* registration of individual T1W and dMRI data (Wu et al., 2018). Then, the transformation was applied to each diffusion-weighted volume, and the gradient vectors were rotated using the rotation matrix estimated from the affine transformation.

### NODDI-based microstructural modeling

To investigate advanced brain microstructural properties, we fit our NODDI model (Zhang et al., 2012) to the multi-shell (i.e., *b* = 1,000, 2,000, and 3,000 s/mm^2^) dMRI data using the Accelerated Microstructure Imaging *via* Convex Optimization (AMICO) toolbox (Daducci et al., 2015). We also used the AMICO toolbox to significantly speed up the time needed to fit the NODDI model by reformulating the NODDI model as a linear system without sacrificing accuracy. Using NODDI, we calculated ICVF, ODI, and ISO.

Each individual’s T1W images were transformed from anatomically corrected space to diffusion-corrected space *via* two-step linear registration using the Functional Magnetic Resonance Imaging of the Brain (FMRIB) Linear Image Registration Tool (FLIRT) (Jenkinson et al., 2012). Advanced Normalization Tools (ANTs)^1^ was used to linearly register each subject’s T1W images to MNI atlas [International Consortium for Brain Mapping (ICBM) 2009b Nonlinear Asymmetric], and the transformation matrix that resulted was applied to warp each subject’s microstructural map to the MNI space. To reduce the effect of fine-grained local variations in anatomy between individuals, voxels within the mask region were then convolved with the specified full-width half maximum (FWHM, equaling voxel size × 2.3548 mm) Gaussian smoothing kernel.

### Statistical analysis

We determined differences in NODDI metrics between RW and SD using a voxel-wise paired *t-*test, with statistical significance set at corrected *P* < 0.05. The false discovery rate (FDR) was used for multiple-comparison corrections. We identified the association between NODDI metrics and VSM performance by calculating the Spearman correlation coefficient using Statistical Product and Service Solutions (SPSS) software version 22.0 (IBM Corp., Armonk, NY, USA).

## Results

Subjects took longer to complete the delayed-CFT trial after SD than after RW (84.6 ± 37.2 *vs.* 67.4 ± 28.2 s; *P* = 0.022). In addition, their scores in the delayed-CFT trial were lower after SD than after RW (22.1 ± 6.9 *vs.* 24.3 ± 7.9; *P* = 0.034). RW and SD states did not differ significantly in their CFT-copy (times: 136.6 ± 43.5 *vs.* 133.3 ± 45.1 s; *P* = 0.788; score: 35.2 ± 1.1 *vs.* 34.9 ± 1.8; *P* = 0.421) or immediate-CFT (times: 108.4 ± 41.2 *vs.* 122.5 ± 48.8 s; *P* = 0.272; score: 24.5 ± 8.6 *vs.* 22.9 ± 7.3; *P* = 0.170) trial results.

**FIGURE 2:**
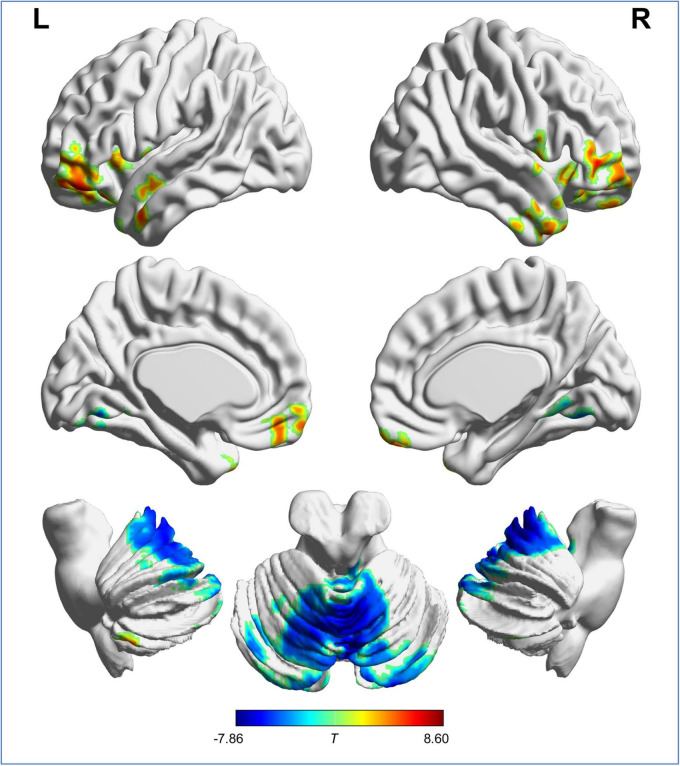
Brain regions with significant difference in ISO between RW and SD. “L” and “R” represent the left and right side of the brain, respectively. Red indicates ISO reduction and blue indicates ISO increment after SD. ISO, isotropic volume fraction; RW, rested wakefulness; SD, sleep deprivation.

Voxel-wise analysis showed no significant difference in ICVF and ODI between RW and SD. The distribution of brain regions with altered ISO is shown in Figure 2 and Table 1. Compared with after RW, after SD, subjects exhibited decreased ISO in the left superior, middle and inferior frontal gyrus, left medial frontal and rectus gyrus, left superior and middle temporal gyrus, temporal pole, and anterior insula, right middle and inferior frontal gyrus, right middle and inferior temporal gyrus and temporal pole, and bilateral posterior cerebellar lobe; and increased ISO in the bilateral anterior and posterior cerebellar lobe and cerebellar vermis, and lingual gyrus.

**TABLE 1:** Differences in isotropic volume fraction between the rested wakefulness and sleep deprivation.

Regions	Voxels	Brodmann area	MNI coordinates			Peak *T-*value
			*x*	*y*	*z*	
Left superior, middle, and inferior frontal gyrus	1,262	10/11/47	−43	43	−17	8.60
Right middle and inferior temporal gyrus and temporal pole	566	21/20/38	47	11	−41	6.07
Left medial frontal gyrus and rectus gyrus	475	11	7	53	−25	5.44
Right middle and inferior frontal gyrus	1,359	11/47	55	39	3	7.71
Left superior and middle temporal gyrus, temporal pole, and anterior insula	613	22/21/38/13	−59	7	−33	6.82
Left superior temporal gyrus and temporal pole	119	38	−33	19	−45	6.49
Right temporal pole	116	22	51	5	1	5.07
Right posterior cerebellar lobe	187		45	−61	−53	7.39
Left posterior cerebellar lobe	199		−37	−41	−55	5.92
Bilateral anterior and posterior cerebellar lobe and cerebellar vermis, and lingual gyrus	4,133	18	5	−71	−19	−7.86

MNI, Montreal Neurological Institute.

**FIGURE 3:**
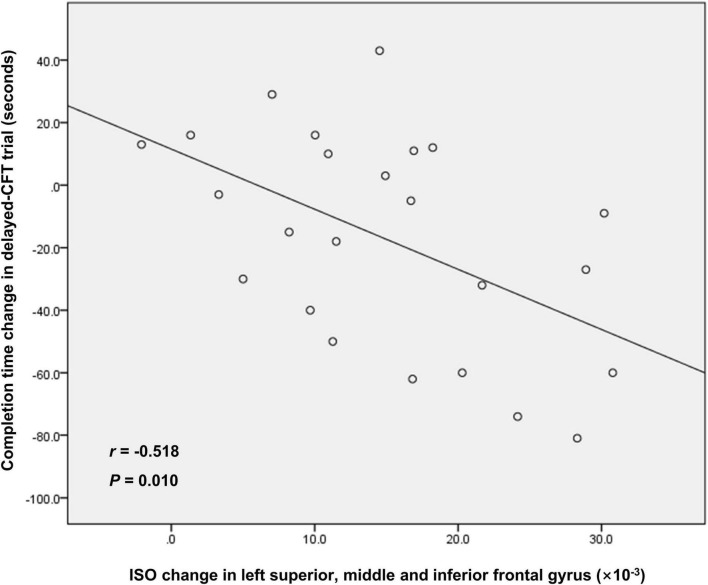
The correlation between ISO change in left superior, middle, and inferior frontal gyrus and completion time change in delayed-CFT trial. ISO, isotropic volume fraction; CFT, complex figure test.

The result of the correlation analysis is shown in Figure 3. Between the RW and SD states, changes in mean ISO in the left superior, middle and inferior frontal gyrus was negatively correlated with changes in completion time in the delayed-CFT trial (*r* = −0.518, *P* = 0.010).

## Discussion

This study aimed to adopt NODDI to explore whether microstructural alterations occurred in GM after SD and determine whether NODDI measurements were associated with VSM. The main findings were as follows. First, we observed no significant changes in ICVF or ODI after SD, indicating that SD hardly affected the density and spatial organization of neurites in GM. Second, ISO decreases were observed in a series of cognition-related brain regions (including the left superior, middle and inferior frontal gyrus, left medial frontal and rectus gyrus, left superior and middle temporal gyrus, temporal pole, and anterior insula, right middle and inferior frontal gyrus, right middle and inferior temporal gyrus and temporal pole) and bilateral posterior cerebellar lobe; while we saw ISO increases in the bilateral anterior and posterior cerebellar lobe and cerebellar vermis, and lingual gyrus. Third, CFT results indicated that SD primarily induced VSM dysfunction, which was in agreement with a previous study (Gosselin et al., 2017). Fourth, after SD, ISO changes in the left superior, middle and inferior frontal gyrus was associated with VSM dysfunction.

Consistent with prior SMT research, which found that SD showed no significant effect on myelin and axonal integrity in WM (as reflected by a lack of differences in intra-neurite volume fraction) (Voldsbekk et al., 2021), our current study revealed that SD hardly affected the density and spatial organization of neurites in GM. Meanwhile, a previous DTI study found that SD alters the content of water molecules in WM, as reflected by decreased mean diffusivity (Ding et al., 2012). A previous SMT study also demonstrated that SD affects the extra-axonal water molecule diffusion process, as shown by a decrease in extra-neurite mean/radial diffusivity (Voldsbekk et al., 2021). In line with these previous studies (Ding et al., 2012; Voldsbekk et al., 2021), the effect of SD on ISO observed in the current study indicated that SD affected the extra-neurite water molecule diffusion process in GM. Given that ISO is suggested to be a biomarker of neuroinflammation (Kraguljac et al., 2022) and that SD leads to neuroinflammation (inducing astrocytic phagocytosis and microglial activation) (Bellesi et al., 2017), the changes in ISO observed in the current study could be attributed to SD-related neuroinflammation processes.

After SD, subjects showed ISO reductions in the prefrontal cortex (including the superior, middle, and inferior frontal gyrus; medial frontal gyrus; and rectus gyrus) and the temporal lobe (including the superior, middle, and inferior temporal gyrus; and temporal pole) and anterior insula. Consistently, as reported in prior studies, SD also causes metabolic activity reduction and functional-communication abnormalities in these brain regions (Thomas et al., 2000; Li et al., 2021). The prefrontal cortex is important for cognitive control, working memory, and behavioral flexibility (Buchta et al., 2017); the temporal lobe is involved in auditory cognition, visual processes, and memory (Jackson et al., 2018). The anterior insula has reciprocal connections to limbic regions (such as the anterior cingulate cortex, ventromedial prefrontal cortex, amygdala, and the ventral striatum), which is involved in the integration of autonomic and visceral information into emotional, cognitive, and motivational functions (Namkung et al., 2017). The decreases in ISO observed in the above-mentioned areas might be one of the neural substrates responsible for SD-related cognitive dysfunctions, such as cognitive control and working memory (Killgore, 2010).

In addition, changes in NODDI metrics after SD in the cerebellum were primarily dominated by ISO increases in the bilateral anterior and posterior cerebellar lobe and cerebellar vermis; and ISO increases were also observed in the bilateral lingual gyrus. In agreement with our findings, a previous study found that SD affects metabolic activity in these regions (Thomas et al., 2000). The anterior cerebellar lobe forms functional circuits with sensorimotor regions to support motor execution, and the posterior cerebellar lobe forms functional circuits with association cortices to support various functions from motor planning to working memory (Stoodley and Schmahmann, 2018). Functionally, the cerebellar vermis is concerned with axial motor control and the modulation of affective behavior (Klein et al., 2016; Fujita et al., 2020). The lingual gyrus is associated with basic visual processing (Palejwala et al., 2020). It has been confirmed that several domains including mood, working memory, motor performance, and visual perception are particularly vulnerable to SD (Durmer and Dinges, 2005; Killgore, 2010), which might be explained by the ISO changes in the cerebellum and lingual gyrus.

Several limitations of the current study should be noted. First is the relatively small sample size; subsequent studies should be performed with larger sample sizes to strengthen statistical significance. Second, we enrolled only female participants to avoid the effect of gender as a confounding factor (Dai et al., 2012) on the results. Third, in this study, we evaluated the effect of SD on visuospatial ability only. SD has been verified to cause detrimental effects across multiple cognitive domains, such as attention, vigilance, perception, memory, and executive functions (Killgore, 2010). The association between NODDI metric alterations and changes in multiple cognitive domains need to be examined in future studies.

In conclusion, our results suggested that SD hardly affected the density and spatial organization of neurites in GM, but it did affect the extra-neurite water molecule diffusion process (perhaps resulting from neuroinflammation), which contributed to VSM dysfunction.

## Data availability statement

The original contributions presented in this study are included in the article/supplementary material, further inquiries can be directed to the corresponding authors.

## Ethics statement

The studies involving human participants were reviewed and approved by the Ethics Committee of Fujian Medical University Union Hospital. The patients/participants provided their written informed consent to participate in this study.

## Author contributions

J-HL: data curation, formal analysis, investigation, and writing – review and editing. X-HC and Y-BC: data curation, formal analysis, investigation, and writing – original draft. YW: methodology. H-JC: conceptualization, data curation, formal analysis, funding acquisition, investigation, project administration, supervision, visualization, and writing – review and editing. N-XH: data curation, formal analysis, funding acquisition, investigation, visualization, and writing – review and editing. All authors contributed to the article and approved the submitted version.

## Footnotes

1
https://stnava.github.io/ANTs
